# Aspartate aminotransferase to platelet ratio correlates with poor prognosis and metabolic alterations in *Dabie bandavirus* infection

**DOI:** 10.3389/fimmu.2024.1471511

**Published:** 2025-01-17

**Authors:** Chunxia Guo, Ruixue Li, Xia Wang, Xiulan Peng

**Affiliations:** ^1^ Department of Infectious Diseases, Union Hospital, Tongji Medical College, Huazhong University of Science and Technology, Wuhan, Hubei, China; ^2^ Department of Internal Medicine, The Hospital of Wuhan University, Wuhan, Hubei, China; ^3^ Department of Pharmacy, The Second Affiliated Hospital of Jianghan University, Wuhan, Hubei, China; ^4^ Department of Oncology, The Second Affiliated Hospital of Jianghan University, Wuhan, Hubei, China

**Keywords:** severe fever with thrombocytopenia syndrome, aspartate aminotransferase to platelet ratio index, metabolic analysis, prognostic biomarker, *Dabie bandavirus*

## Abstract

**Introduction:**

Severe fever with thrombocytopenia syndrome (SFTS) is an emerging infectious disease with a high mortality rate caused by *Dabie bandavirus*. The aspartate aminotransferase to platelet ratio index (APRI) is a biomarker of liver injury and inflammation. This study aimed to examine the correlation between APRI and SFTS prognosis using clinical data analysis and attempt to explain its prognostic significance through metabolic analysis.

**Methods:**

Data from hospitalized patients with a confirmed diagnosis of SFTS virus infection at Wuhan Union Hospital were retrospectively collected. The low and high APRI groups were 1:1 matched using propensity score matching (PSM) analysis. Fresh plasma was collected from patients with SFTS on admission and used for metabolic tests.

**Results:**

A total of 617 patients with SFTS who met the inclusion criteria were selected for analysis. Survival analysis revealed that patients with SFTS with high APRI (> 35.3) had a substantially higher death rate than those with low APRI (≤ 35.3). Receiver operating characteristic analysis showed the predictive performance of APRI for SFTS prognosis is 0.77, with a 95% CI of 0.73–0.80, which was superior to NLR (area under the curve (AUC): 0.65), platelet-to-lymphocyte ratio (AUC: 0.54), and systemic immune-inflammation index (AUC: 0.58). The prognostic value and predictive performance of APRI were more substantial after PSM than before PSM. Metabolomic testing identified several differential serum metabolites, with alanine, aspartate, glutamate, glycerophospholipid, and tryptophan metabolism being the most important metabolic pathways.

**Conclusion:**

A high APRI score was associated with relatively higher mortality in patients with SFTS, and its predictive performance for the survival outcome of SFTS was superior to that of well-recognized inflammatory scores. Alanine, aspartate, and glutamate metabolism are involved in the progression of SFTS.

## 1 Introduction

Severe fever with thrombocytopenia syndrome (SFTS) is an emerging infectious disease caused by the SFTS virus (SFTSV), now known as the *Dabie bandavirus (* 1, 2). Recently, a person-to-person transmission cluster of SFTS was reported, characterized by mixed SFTSV infection with both familial and nosocomial clustering (3). SFTS was first identified in China in 2009 (4), with a growing number of cases subsequently reported in Japan (5) and Korea (6). Typical clinical manifestations of SFTS include high fever, myalgia, severe thrombocytopenia, digestive symptoms, and even irritation (7). Common complications include acute pancreatitis, viral myocarditis, invasive pulmonary aspergillosis, hemophagocytic lymphohistiocytosis (HLH), and toxic encephalopathy. The progression of severe SFTS is usually rapid, with a relatively high mortality rate of 10%–30% (8). With substantial global ecological and climate changes, the rapid spread of SFTSV has become a serious public health concern in Asia (9).

The underlying mechanism of SFTS is characterized by a systemic inflammatory response syndrome (SIRS), which is induced by viral replication and viremia. SIRS can cause dysfunction in multiple organs through a variety of cytokines. A previous study showed that IL-6 and IL-10 levels were strongly associated with the outcomes of patients with SFTSV infection (10). Moreover, activating NF-κB pathway could facilitate the replication of SFTSV in human monocytes (11). Some patients with SFTS developed HLH, a progressive systemic inflammatory disease characterized by excessive cytokine production and cytopenia (12). In our previous study (13), we assessed the prognostic significance of the ratio of C-reactive protein (CRP) to the prognostic nutritional index and found a strong correlation with disease type and survival outcomes in patients with SFTS. Given the essential role of the systemic inflammatory response in the process of SFTS, there is an urgent need to design and validate a novel inflammatory score for the prognostic assessment of patients with SFTS.

In addition to decreased platelet count, patients with SFTS are prone to liver injury (14). Aspartate aminotransferase (AST) levels in SFTS are usually higher than those of alanine aminotransferase (ALT), and AST reflects mitochondrial impairment with sensitively (15). The AST to platelet ratio index (APRI) has been used in clinical research to predict liver cirrhosis (16), liver failure (17), and liver cancer (18). Recently, APRI has been used to predict survival outcomes in hemorrhagic fever with renal syndrome (HFRS), with the APRI at admission serving as a reliable biomarker to identify patients at high risk of death from HFRS (19). Assessment of the APRI score is quite easy and involves indices from routine blood tests and liver function, which are part of the routine tests and are cost-effective for those patients. Currently, there is no literature discussing the role of APRI in SFTS, and our work will fill this gap and provide an important reference for the risk assessment of patients with SFTS.

In view of the clinical features of SFTS, we hypothesize that APRI measured at the early stage of SFTS may be potentially utilized to predict the survival outcome of SFTS individuals. Therefore, in the present study, we initially determined whether APRI is the independent risk factor of SFTS patients. Then we compared the predictive performance of APRI with other inflammatory scores, such as neutrophil-to-lymphocyte ratio (NLR), platelet-to-lymphocyte ratio (PLR) and systemic immune-inflammation index (SII). Finally, we analyzed the metabolic alterations between SFTS individuals with low and patients with high APRI, to expound the metabolic changes caused by dysregulation of APRI in SFTS individuals.

## 2 Methods

### 2.1 Study design and population

A retrospective cohort study from Jan. 2022 to Jun. 2024 related to SFTS was undertaken in Wuhan Union Hospital, which is a regional center of diagnosing and treating SFTS. The diagnosis and therapeutic schedule of SFTS was performed in accordance with the 2022 guideline of SFTS issued by Chinese Infectious disease Association (20). The inclusion criteria were: (a) patients with confirmed diagnosis of SFTSV infection detected by Reverse Transcription-Polymerase Chain Reaction (RT-PCR). (b) SFTS individuals whose age over 18 years old. The exclusion criteria were: (a) SFTS patients with cirrhosis and splenic hyperfunction; (b) SFTS patients with hematological system diseases; (c) SFTS patients with critical information missing. The clinical research related to SFTS was reviewed and then approved by the Ethics Committee of Union hospital (No.2024-0611), and this research was implemented in line with the main principles of Helsinki Declaration. The informed consent was obtained from all the SFTS participants.

### 2.2 Data collection

The demographic data, treatment information, and clinical outcomes of patients with SFTS were retrospectively obtained from the medical system. Clinical information included age, sex, routine blood tests, SFTSV load, and liver and renal function. The disease type of patients with SFTS was categorized into mild, moderate, severe, and critical types according to the SFTS guidelines issued by the Chinese Infectious disease Association (20). For patients with severe or critical SFTS who discontinued treatment and returned home, we conducted a follow-up telephone interview to ensure the final outcome.

We selected lymphocytes (LYM), platelets (PLT), neutrophils (Neu), white blood cells, and hemoglobin from routine blood samples for subsequent analysis. Biochemical tests included ALT, total protein, total bile acid, AST, blood urea nitrogen, albumin, creatinine, glucose, and creatine kinase. Serum inflammatory indices included procalcitonin and CRP. Coagulation function included activated partial thromboplastin time (APTT), prothrombin time (PT), D-dimer levels. The combined scores were defined as follows: APRI = AST(U/L)/PLT(109/L)×100 (19), neutrophil-to-lymphocyte ratio (NLR) = Neu(10^9^/L)/LYM(10^9^/L), platelet-to-lymphocyte ratio (PLR) = PLT(10^9^/L)/LYM(10^9^/L), and systemic immune-inflammation index (SII) = PLT(10^9^/L)×Neu(10^9^/L)/LYM(10^9^/L). The cut-off values for the single indices from routine blood tests, biochemical testing, and coagulation function were determined by their lower or upper limits in our hospital, as defined in our previous study (13). The cut-off values of APRI, NLR, PLR, and SII were automatically determined using X-tile software (version 3.3.2).

### 2.3 Metabolite detection

The blood samples, previously collected for virus retest were used for the current analysis. The fresh blood samples were collected from 37 patients with SFTS admitted to our hospital during the acute phase. The blood samples were divided into serum and peripheral blood mononuclear cells (PBMCs) via centrifugation in the local laboratory. Both the plasma and PBMCs were immediately frozen and stored at −80°C until use. Liquid chromatography-tandem mass spectrometry analyses of plasma were conducted using an ultra high-performance liquid chromatography system (Agilent Technologies, Santa Clara, California, USA). Detailed procedures for these analyses have been reported in a recent study (21). Orthogonal partial least squares discriminant analysis (PLS-DA) was conducted through Major platform via the R package “ropls”(version 1.6.2). Substantial metabolites between the low and high APRI groups were identified based on variable importance in the projection values. Differential metabolic pathways between the low and high APRI groups were searched using the Kyoto Encyclopedia of Genes and Genomes database (KEGG) database.

### 2.4 Statistical analysis

For continuous data, the differences between the low and high APRI groups were measured via the sample t test or Mann-Whitney U test, as appropriate. Categorical data between the low and high APRI groups were compared using either X^2^ test or Fisher’s exact test. We conducted rigorous 1:1 propensity score matching (PSM) analysis to adjust the differences at the baseline clinical features between the low and high APRI groups. The PSM method could effectively reduce the potential confounding and selection bias in the observational research. The independent risk factors with relevance to the in-hospital survival outcomes of SFTS individuals were determined by LASSO regression analysis. Kaplan-Meier curves combined with log-rank test were utilized to compare the mortality rate between the low and high APRI groups. The receiver operating characteristic (ROC) curves were generated to assess the predictive values of the combined scores (APRI, NLR, PLR and SII), and the area under the curve (AUC) with 95% confidence interval (CI) was quantified for comparison via DeLong test. Statistical analysis and data processing were performed in SPSS software (version 21.0) and R software (version 4.1.0). Statistical significance in our analyses was deemed as two-sided P -value less than 0.05.

## 3 Results

### 3.1 Clinical characteristics of patients with SFTS

In total, 617 patients with SFTS who met the inclusion criteria were enrolled in this study. The mean age of these patients was 61.45 years, with a median age of 62 years. Among them, 85 patients died during hospitalization. All participants were from rural or mountainous villages. The study included 261 male and 356 female patients. According to the 2010 edition of the SFTS guidelines, 60 cases were mild, 209 were moderate, 213 were severe, and 135 were critical.

### 3.2 Correlation between APRI and prognosis

**Figure 1:**
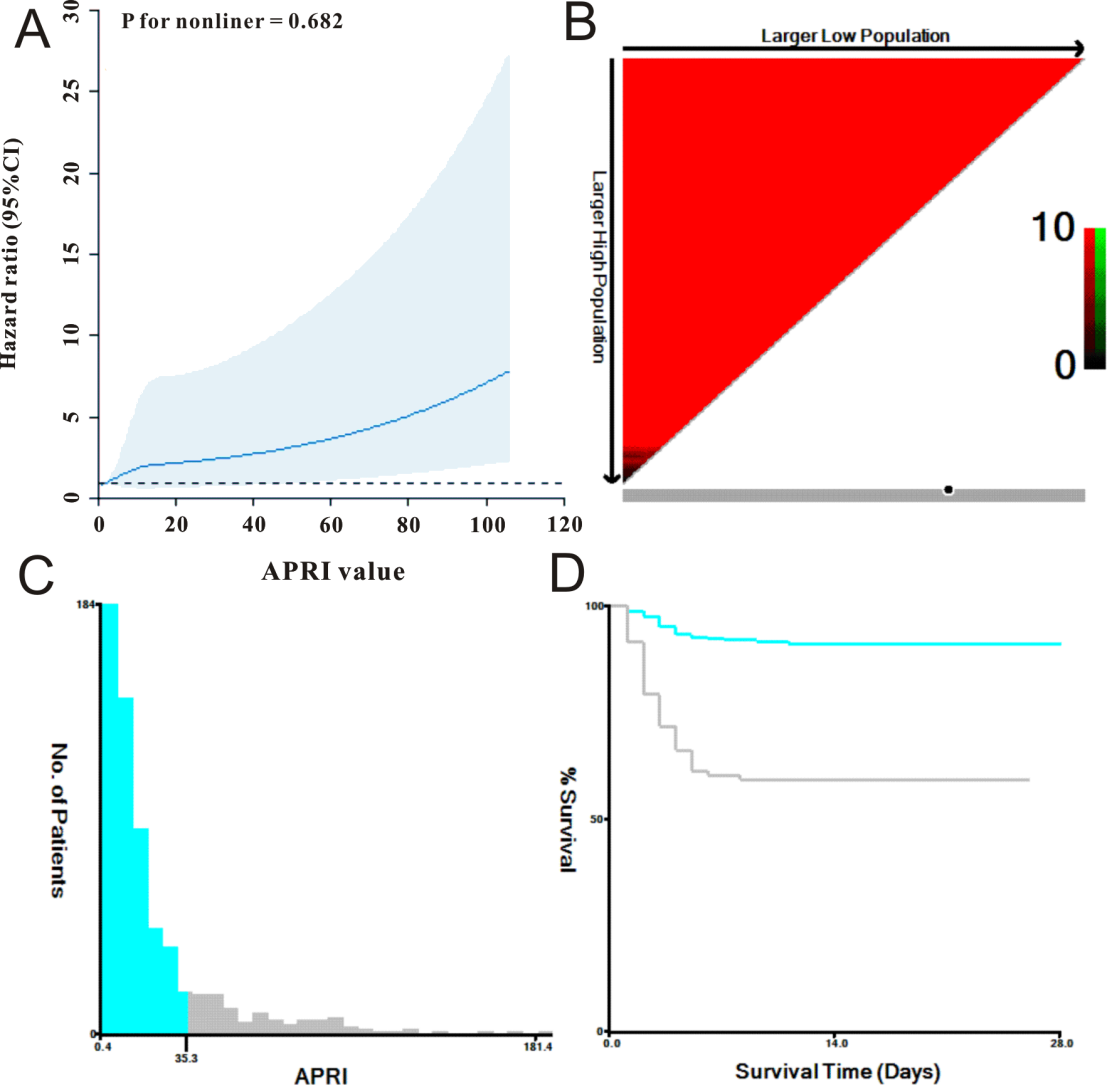
The selection of best cut-off threshold of APRI. **(A)** APRI was shown to be nonlinear with mortality of SFTS individuals using restricted cubic spline model. **(B, C)** The distribution of APRI with sample size of SFTS individuals. **(D)** Survival curve of APRI with the cut-off value being 35.3.

First, we utilized a restricted cubic spline to visualize the relationship between APRI distribution and mortality in patients with SFTS. As shown in **Figure 1A**, the relationship between APRI and mortality was nonlinear (P = 0.682). We then determined the optimal APRI cut-off using X-tile software. As shown in **Figures 1B–D**, when the threshold of APRI was set at 35.3, the risk stratification for survival outcome of patients with SFTS was most remarkable. Based on the optimal APRI threshold, we divided the 617 patients with SFTS into low APRI (N = 510) and high APRI (N = 107) groups. As shown in **Table 1**, the differences in age, disease type, sex, and most biochemical indicators between the two groups were statistically significant (p < 0.05). Due to the unbalanced status between the low and high APRI groups, we utilized propensity score matching (PSM) analysis to solve this issue. After adjustment for age, disease type and gender, the distributions of most biochemical indicators were balanced between the low APRI group (N=98) and high APRI group (N=98), which is also listed in **Table 1**.

**Table 1:** Comparisons of baseline characteristics between original cohort and matched cohort.

Characteristics	Original cohort (n=617)			Matched cohort (n=196)		
	Low APRI (n=510)	High APRI (n=107)	P value	Low APRI (n=98)	High APRI (n=98)	P value
Age, years old	60.7 ± 10.7	65.3 ± 8.5	<0.001	64.1 ± 8.5	65.3 ± 8.6	0.336
Gender, male, n (%)	218 (42.7)	43 (40.2)	0.704	42 (42.9)	41 (41.8)	1.000
Disease type, n (%)			<0.001			1.000
Mild	60 (11.8)	0 (0.0)		0 (0.0)	0 (0.0)	
Moderate	202 (39.6)	7 (6.5)		7 (7.1)	7 (7.1)	
Severe	185 (36.3)	28 (26.2)		28 (28.6)	28 (28.6)	
Critical	63 (12.4)	72 (67.3)		63 (64.3)	63 (64.3)	
Secondary infection			0.933			0.144
Yes	76 (14.9)	15 (14.0)		23 (23.5)	14 (14.3)	
No	434 (85.1)	92 (86.0)		75 (76.5)	84 (85.7)	
Viral load, n (%)			0.030			1.000
Low	118 (23.1)	14 (13.1)		13 (13.3)	14 (14.3)	
High	392 (76.9)	56 (86.9)		85 (86.7)	84 (85.7)	
Laboratory values						
WBC, × 10^9^/L	3.6 ± 0.8	3.6 ± 1.5	0.983	3.6 ± 1.2	3.5 ± 1.5	0.667
Hemoglobin, g/L	127.0 ± 17.8	127.1 ± 19.4	0.953	124.0 ± 19.1	128.4 ± 18.7	0.107
Platelet, × 10^9^/L	54.3 ± 21.4	29.8 ± 13.6	<0.001	33.5 ± 16.2	30.1 ± 13.0	0.113
NEU, × 10^9^/L	2.4 ± 1.0	2.3 ± 1.1	0.633	2.5 ± 0.9	2.2 ± 0.8	0.156
LYM, × 10^9^/L	0.9 ± 0.5	0.9 ± 0.4	0.331	0.8 ± 0.4	0.9 ± 0.5	0.156
AST, U/L	165.5 (91.8, 277.3)	596.0 (409.0, 778.0)	<0.001	197.0 (124.0, 283.0)	613.5 (782.8, 613.5)	<0.001
ALT, U/L	68.5 (44.0, 113.0)	171.0 (126.0, 253.0)	<0.001	68.5 (49.8, 112.5)	177.0 (126.0, 253.0)	<0.001
ALP, U/L	79.6 ± 28.7	120.5 ± 56.5	<0.001	89.0 ± 39.6	121.4 ± 56.7	0.008
TBIL, umol/L	10.7 ± 3.8	14.3 ± 5.5	<0.001	12.1 ± 4.1	14.1 ± 5.4	0.150
TP, g/L	57.8 ± 6.2	55.8 ± 6.3	0.003	55.8 ± 6.4	55.9 ± 6.5	0.878
Albumin, g/L	32.2 ± 4.5	29.3 ± 4.0	<0.001	30.5 ± 4.8	29.4 ± 4.1	0.074
BUN, umol/L	5.3 ± 2.3	7.1 ± 3.2	<0.001	6.3 ± 2.6	7.0 ± 3.3	0.309
Creatinine, umol/L	76.5 ± 36.8	96.9 ± 47.0	<0.001	82.6 ± 36.5	93.4 ± 42.3	0.218
Glucose, mmol/L	7.0 ± 2.8	8.3 ± 3.7	<0.001	7.7 ± 3.1	8.3 ± 3.8	0.246
LDH, U/L	714.5 (472.8, 1027.0)	1784.0 (1195.0, 2337.0)	<0.001	882.5 (631.8, 1288.9)	1782.5 (1189.0, 2340.0)	<0.001
CK, U/L	451.0 (218.3, 1129.8)	1162.0 (497.0, 2623.0)	<0.001	683.0 (332.8, 1408.3)	1782.5 (1189.0, 2340.0)	<0.001
APTT, s	52.4 ± 15.8	70.6 ± 24.5	<0.001	58.0 ± 24.1	71.2 ± 24.4	<0.001
PT, s	13.0 ± 1.3	13.7 ± 1.7	<0.001	13.3 ± 2.1	13.7 ± 1.5	0.154
D dimer	4.2 ± 1.5	7.4 ± 3.8	<0.001	5.4 ± 2.2	7.5 ± 3.7	0.008
PCT	0.8 ± 0.4	1.3 ± 0.6	0.059	0.9 ± 0.4	1.2 ± 0.6	0.231
Hs-CRP	10.0 ± 4.5	13.1 ± 6.1	0.118	13.6 ± 6.6	11.0 ± 5.0	0.277
Outcomes						
Time, days	9.0 (7.0, 12.0)	9.0 (3.0, 12.0)	0.003	11.0 (7.0, 15.0)	9.0 (3.0, 12.0)	0.007
Death, n (%)	42 (8.2)	43 (40.2)	<0.001	21 (21.4)	38 (38.8)	0.013

APRI, aspartate aminotransferase to platelet ratio index; WBC, white blood cells; NEU, neutrophil; LYM, lymphocyte; AST, aspartate aminotransferase; ALT, alanine aminotransferase; ALP, alkaline phosphatase; TBIL, total bilirubin; LDH, lactate dehydrogenase; CK, creatine kinase; APTT, activated partial thromboplastin time; PT, prothrombin time; PCT, procalcitonin; Hs-CRP, high-sensitivity C-reactive protein.

**Figure 2:**
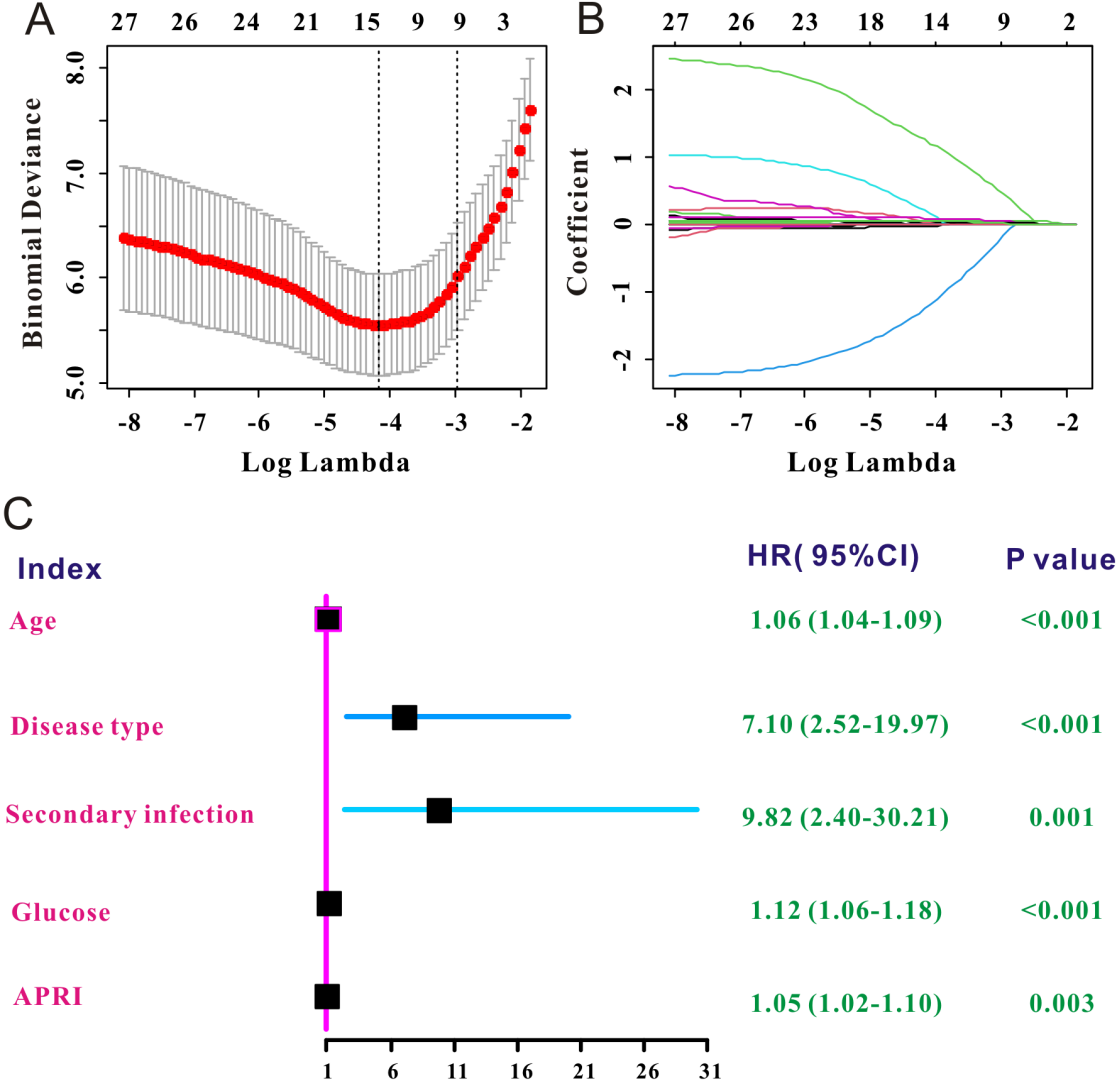
Independent prognostic factors of SFTS selected by LASSO Cox. **(A)** The selection process of the optimum threshold of the λ in the Lasso Cox regression model. **(B)** The variation characteristics of the coefficient of the included clinical variables. **(C)** Forest plot of the independent prognostic factors of SFTS.

LASSO Cox regression was used to select the prognostic factors for SFTS. As shown in **Figures 2A, B**, nine clinical features, including age, disease type, secondary infection, creatinine, lactate dehydrogenase, glucose, APTT, PT, and APRI, were selected for subsequent prognostic assessment (**Table 2**). Further multivariate Cox regression indicated that high level of APRI was an independent factor (hazard ration (HR) = 1.05, 95% confidence interval (CI): 1.02–1.10, P = 0.003) for worse prognosis of SFTS (**Figure 2C**).

**Table 2:** Univariate and multivariate COX analysis for in-hospital mortality selected by LASSO regression.

	Univariate		Multivariate	
	HR (95% CI)	P	HR (95% CI)	P
Age	1.07 (1.05-1.10)	<0.001	1.06 (1.04-1.09)	<0.001
Disease severity				
Mild/moderate	Ref.	–	Ref.	–
Severe/critical	16.99 (6.23-46.37)	<0.001	7.10 (2.52-19.97)	<0.001
Secondary infection				
Yes	7.93 (1.95-32.26)	0.004	9.82 (2.40-30.21)	0.001
No	Ref.	–	Ref.	–
Creatinine	1.01 (1.01-1.10)	<0.001	1.01 (0.99-1.04)	0.076
Lg(LDH)	15.41 (8.44-28.14)	<0.001	2.05 (0.90-4.69)	0.088
Glucose	1.19 (1.14-1.25)	<0.001	1.12 (1.06-1.18)	<0.001
APTT	1.03 (1.03-1.04)	<0.001	1.01 (0.98-1.04)	0.107
PT	1.32 (1.23-1.41)	<0.001	1.10 (0.98-1.23)	0.093
APRI	1.08 (1.03-1.16)	<0.001	1.05 (1.02-1.10)	0.003

LASSO, least absolute shrinkage and selection operator; HR, hazard ratio; 95%CI, 95% confidence index; LDH, lactate dehydrogenase; APTT, activated partial thromboplastin time; PT, prothrombin time; APRI, aspartate aminotransferase to platelet ratio index.

**Figure 3:**
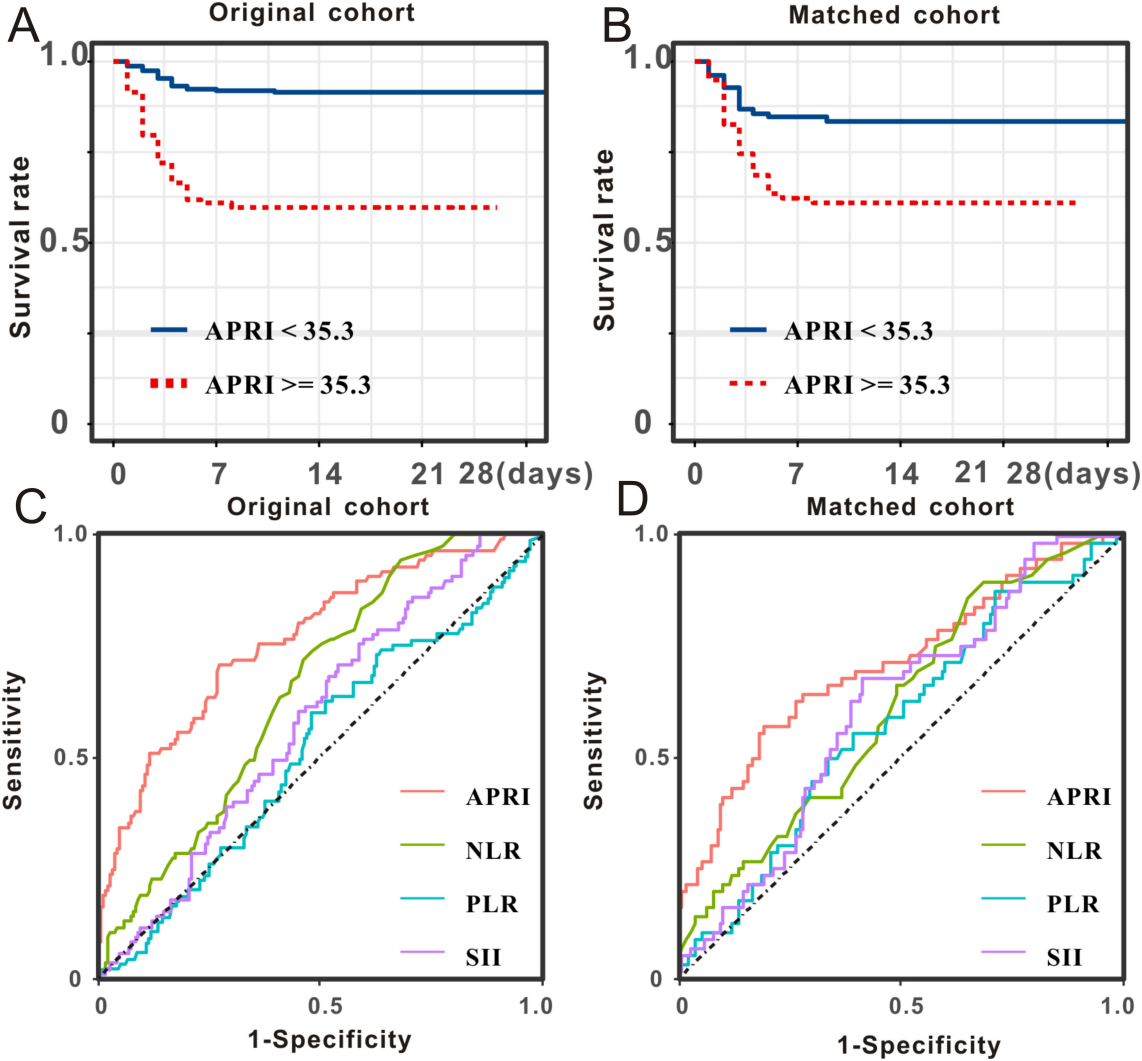
Prognostic significance and predictive performance of APRI for SFTS individuals. SFTS individuals with low levels of APRI experienced better survival outcome than those with high levels of APRI both in the primary cohort **(A)** and PSM cohort **(B)**. Predictive performances of APRI and common inflammatory scores measured by ROC curves in the primary cohort **(C)** and PSM cohort **(D)**.

The Kaplan-Meier survival analysis in the primary cohort revealed that patients with SFTS with high APRI (> 35.3) had a substantially higher death rate than those with low APRI (≤ 35.3, **Figure 3A**). The prognostic value of APRI in the PSM cohort (HR = 2.68, 95% CI: 1.49–4.81, P < 0.001, **Figure 3B**) was also significant. After adjusting for age, sex, and disease severity, the prognostic significance of APRI remained robust (**Table 3**). After further adjustment for viral load, secondary infection, and laboratory results, the prognostic significance of APRI remained robust (**Table 3**). Subsequently, receiver operating characteristic (ROC) analysis determined that the predictive performance of APR for SFTS prognosis was 0.77, with a 95% CI of 0.73–0.80 (**Figure 3C**). When reanalyzing the ROC in the PSM cohort, the area under curve AUC was 0.71, with a 95% CI of 0.64–0.77 (**Figure 3D**). In brief, the APRI is a reliable prognostic biomarker for SFTS.

**Table 3:** Summary of the low and high APRI groups for clinical outcomes before and after PSM.

Methods	In-hospital mortality	
	HR (95%CI)	P value
Cox proportional hazards model	5.17 (3.60-8.14)	<0.001
Cox proportional hazards model with adjust I	3.45 (2.08-6.88)	<0.001
Cox proportional hazards model with adjust II	1.92 (1.06-3.48)	0.033
Propensity score matching	2.68 (1.49-4.81)	<0.001
Propensity score matching with adjust I	2.23 (1.23-4.05)	0.008
Propensity score matching with adjust II	1.79 (1.07-4.15)	0.031

APRI, aspartate aminotransferase to platelet ratio index; PSM, propensity score matching; HR, hazard ratio; 95%CI, 95% confidence index; Adjust I model adjusted for age, gender, disease severity, Adjust II model adjusted for adjust I model plus viral load, secondary infection, and laboratory results.

### 3.3 Comparison of predictive performance via ROC analysis

**Figure 4:**
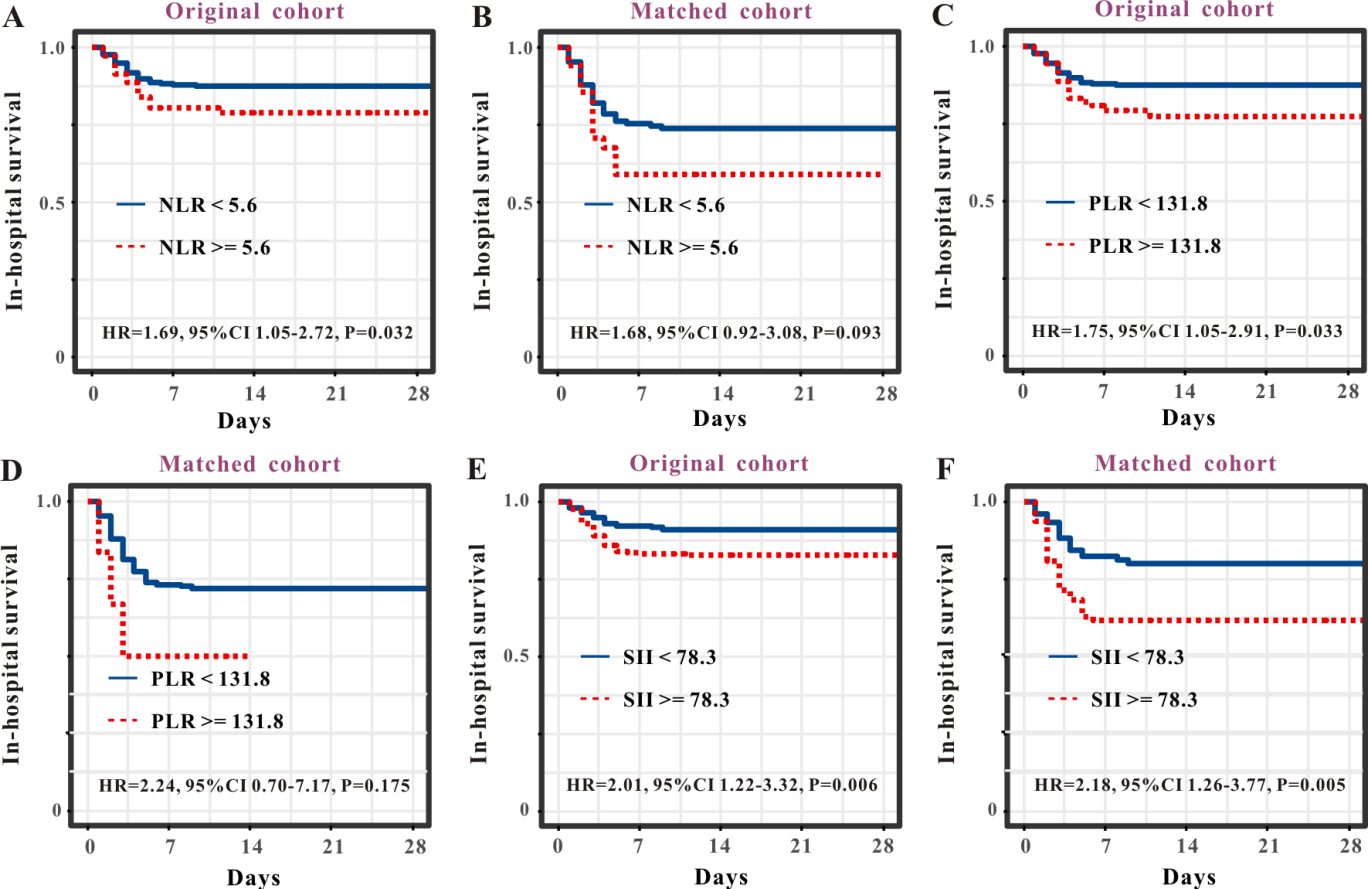
Survival curves of SFTS patients stratified by NLR, PLR and SII. Survival curves of NLR for the prognosis of SFTS individuals from the primary cohort **(A)** and PSM cohort **(B)**. Survival curves of PLR for the prognosis of SFTS individuals from the primary cohort **(C)** and PSM cohort **(D)**. Survival curves of SII for the prognosis of SFTS individuals from the primary cohort **(E)** and PSM cohort **(F)**.

As the NLR, PLR, and SII are well-recognized inflammatory indices, we performed comparative analyses. The survival curve displayed that patients with SFTS and high levels of NLR (> 2.3) experienced shorter survival times than those with low levels of NLR (≤ 2.3) in both the primary cohort (**Figure 4A**) and PSM cohort (**Figure 4B**). Subsequent ROC analysis determined that the predictive performance of the NLR for SFTS prognosis was 0.65, with a 95% CI of 0.62–0.69 (**Figure 3C**). When reanalyzing the ROC in the PSM cohort, the AUC was 0.61, with a 95% CI of 0.54–0.68 (**Figure 3D**). The survival curve displayed that patients with SFTS and high levels of PLR (> 26.3) experienced shorter survival time than those with low levels of NLR (≤ 26.3) in both the primary cohort (**Figure 4C**) and PSM cohort (**Figure 4D**). ROC analysis determined the predictive performance of PLR for SFTS prognosis was 0.54, with a 95% CI of 0.49–0.58 (**Figure 3C**). When reanalyzing the ROC in the PSM cohort, the AUC was 0.58, with a 95% CI of 0.51–0.65 (**Figure 3D**). The survival curve displayed that patients with SFTS and high levels of SII (> 78.3) experienced shorter survival time than those with low levels of SII (≤ 78.3) in both the primary cohort (**Figure 4E**) and PSM cohort (**Figure 4F**). ROC analysis revealed that the predictive performance of the SII for SFTS prognosis was 0.58, with a 95% CI of 0.54–0.62 (**Figure 3C**). When reanalyzing the ROC in the PSM cohort, the AUC was 0.61, with a 95% CI of 0.54–0.68 (**Figure 3D**). As shown in **Table 4**, APRI had the best predictive value for the prognosis of SFTS.

**Table 4:** The comparison of ARPI and other markers for in-hospital mortality before and after PSM.

Markers	Youden index	AUC (95%CI)	P value^1^	Sensitivity	Specificity	PPV	NPV
Original cohort							
APRI	0.433	0.77 (0.73-0.80)	–	70.6	72.7	2.6	0.4
NLR	0.260	0.65 (0.62-0.69)	0.008	72.1	52.9	1.5	0.5
PLR	0.101	0.54 (0.49-0.58)	<0.001	86.5	33.5	1.1	0.3
SII	0.165	0.58 (0.54-0.62)	<0.001	76.5	40.0	1.3	0.6
Matched cohort							
APRI	0.389	0.71 (0.64-0.77)	–	67.1	70.7	1.6	0.6
NLR	0.207	0.61 (0.54-0.68)	0.109	89.3	31.4	1.3	0.3
PLR	0.164	0.58 (0.51-0.65)	0.048	50.0	66.4	1.5	0.8
SII	0.264	0.61 (0.54-0.68)	0.121	67.9	58.6	1.6	0.6

^1^compared with APRI by DeLong test. APRI, aspartate aminotransferase to platelet ratio index; PSM, propensity score matching; AUC, area under the curve; 95%CI, 95% confidence index; PPV, positive predictive value; NPV, negative predictive value; NLR, neutrophil to lymphocyte ratio; PLR, platelet to lymphocyte ratio; SII, systemic immune inflammation.

### 3.4 Metabolic analysis

**Figure 5:**
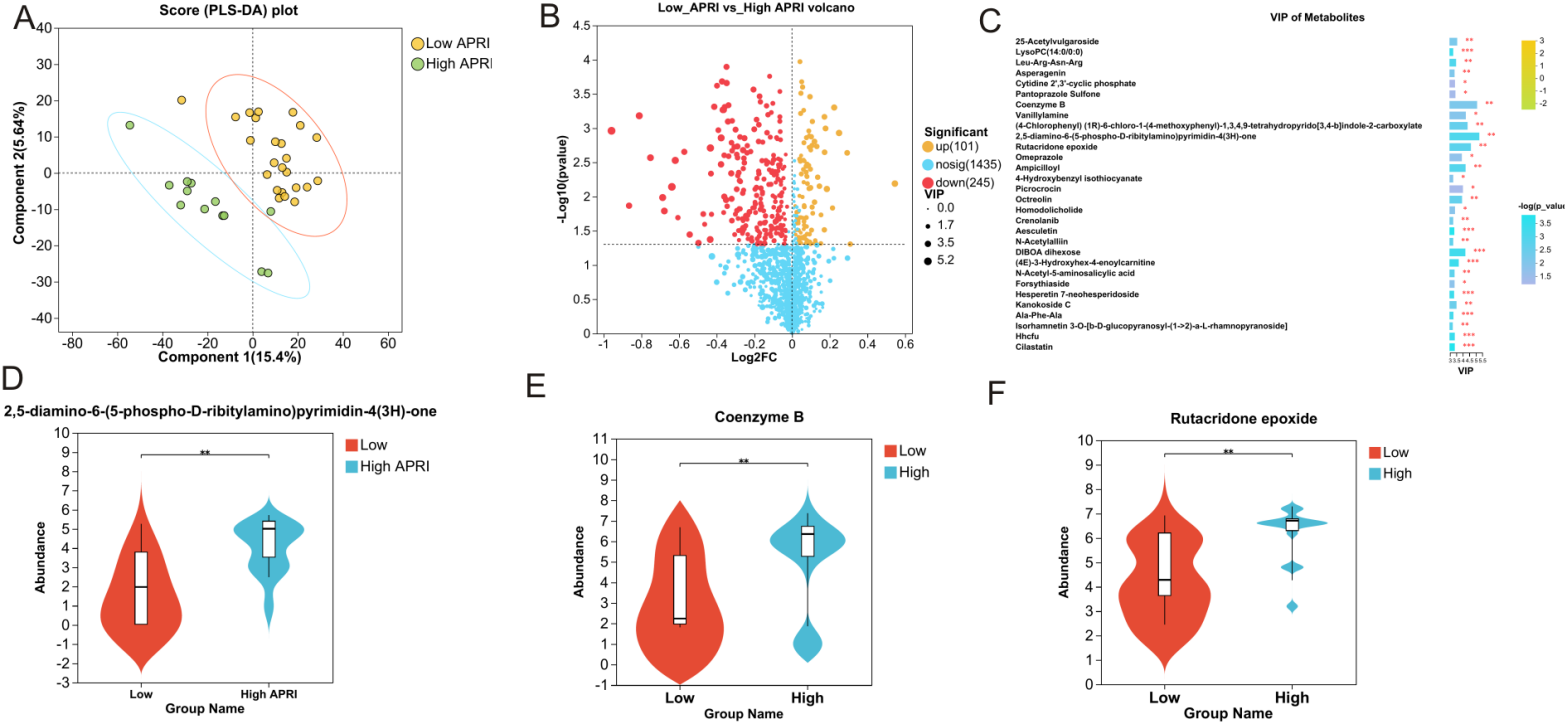
Significant plasma metabolites between the low and high APRI groups. **(A)** The PLS-DA model of the low and high APRI groups. **(B)** Volcano map of the up-regulated and down-regulated plasma metabolites between the low and high APRI groups. **(C)** Significant plasma metabolites based on the VIP value. The expression profiles of 2,5-diamino-6-(5-phospho-D-ribitylam ino)pyrimidin-4(3H)-one **(D)**, Coenzyme B **(E)** and Rutacridone epoxide **(F)** between the low and high APRI groups. ** stands for P<0.01.

**Figure 6:**
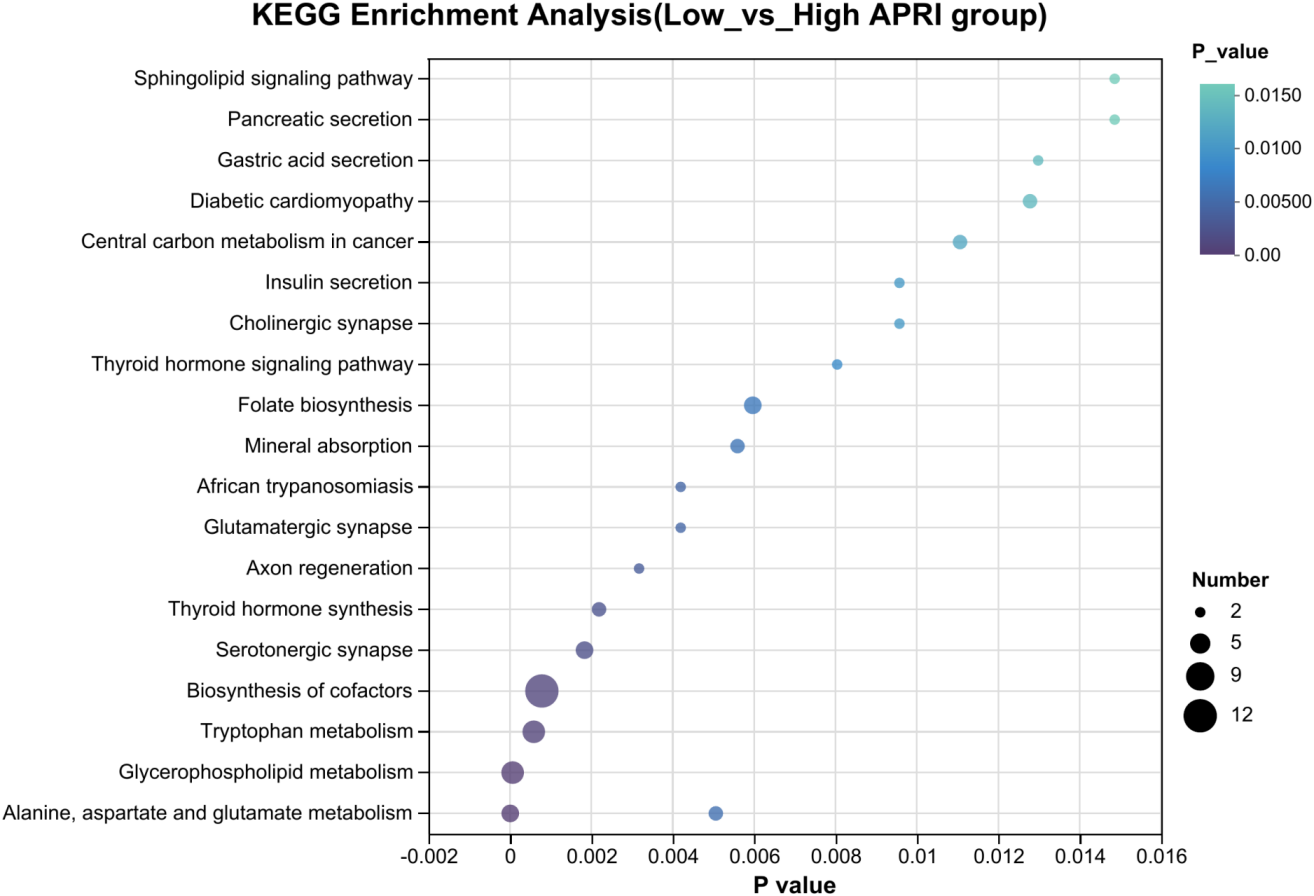
The KEGG pathways showing significant differential plasma metabolites between the low and high APRI groups.

Clear clustering of SFTS cases using the PLS-DA model is shown in **Figure 5A**, where the SFTS samples between the low and high APRI groups were clearly separated. A total of 1,781 metabolites were detected in 37 SFTS blood samples, of which 101 were upregulated and 245 were down-regulated between the low and high APRI groups (**Figure 5B**).Then we utilized the VIP value to determine the significant metabolites between the low and high APRI groups, and we found that 2,5-diamino-6-(5-phospho-D-ribitylamino)pyrimidin-4(3H)-one, coenzyme B and rutacridone epoxide were the top three metabolites between the two groups (**Figure 5C**). The detailed expression profiles of the top three metabolites between the two groups were shown in **Figures 5D–F**. Finally, KEGG analysis was performed to identify the most important biological pathways based on the above significant metabolites. As shown in **Figure 6**, four important differential metabolic pathways were picked out, including alanine, aspartate and glutamate metabolism, glycerophospholipid metabolism, tryptophan metabolism and biosynthesis of cofactors.

## 4 Discussion

Early risk stratification of patients with SFTS will facilitate the optimal use of medical resources. It is important to design a novel serum score for early risk stratification of patients with SFTS. The present study is the first to illustrate the prognostic role of APRI in SFTS and the metabolic changes between low and high APRI groups. Cox regression analysis indicated that a high APRI is a strong risk factor for worse prognosis in the population with SFTS. Subsequent ROC analyses revealed that the performance of the APRI in predicting the survival status of patients with SFTS was better than that of common serum inflammatory scores, such as NLR, PLR, and SII. Our metabolomic analyses identified a group of differential serum metabolites, with alanine, aspartate, glutamate, glycerophospholipid, and tryptophan being the most important metabolic pathways. Hence, our study provides a reliable inflammatory score for prognostic prediction and sheds light on the potential pathogenesis of SFTSV invasion from a metabolomic perspective.

The APRI is a noninvasive score derived from AST and PLT levels. Serum AST is commonly used to evaluate liver tissue damage, while PLT levels correlate with severe infection. Previous studies have demonstrated that both high AST levels and low PLT levels are correlated with less favorable survival outcomes in patients with SFTS. Regarding the APRI score, the correlation between the prognosis of patients with SFTS and serum AST or PLT levels was amplified. Thus, the performance of the APRI score was substantially better in predicting SFTS prognosis than serum AST level or PLT alone. APRI has been demonstrated to be an early warning score for sepsis-associated liver injury (22). The APRI at admission could serve as a reliable biomarker for identifying patients with HFRS who are at high risk of death (19). Moreover, APRI has also been reported to be a reliable marker for identifying high-risk patients with malaria (23). In summary, these findings indicate that the APRI score is suggestive of multiorgan injury in acute infectious diseases. APRI is derived from two routine laboratory parameters (AST and PLT) at a low cost. Hence, our study provides an easy-to-use serum score with high performance in predicting the survival outcome of patients with SFTS using routine blood tests and liver function on admission.

Zhang et al. (24) evaluated the clinical significance and prognostic role of APRI in 131 patients with SFTS and found that those with APRI > 20.37 had a shorter survival time than those with APRI ≤ 20.37, indicating that APRI was associated with unfavorable survival outcomes among the population with SFTS. Unlike their research, we determined the APRI the cutoff value using X-tile instead of ROC. In addition to the clinical analysis based on 617 patients with SFTS, we conducted a metabolic analysis to investigate the metabolic changes caused by dysregulation of the APRI. In this study, we identified the APRI as a reliable inflammatory score for predicting in-hospital mortality in patients with SFTS. Well-established serum inflammatory scores, such as NLR, PLR, and SII, have already been demonstrated to be reflective of the in-hospital survival outcomes of patients with acute infectious diseases. Wang et al. (25) reported that a serum NLR more than 5.4 can significantly increase the risk of death (HR = 6.767, P = 0.011) in patients with SFTS. Wei et al. (26) also reported that the NLR is an efficient early warning score to help medical workers identify patients with SFTS who are at high risk of death. Wang et al. (25) found that PLR was not an independent prognostic factor in patients with SFTS. The SII reflects the systemic inflammatory response, which has been confirmed to be related to the prognosis of patients with cancer and those with acute infectious disease. A recent study with a small sample size revealed no substantial difference in SII between patients with SFTS who died and those who survived; thus, SII is not a reliable prognostic biomarker for SFTS (27). Consistent with the literature, our ROC analyses revealed that the predictive ability of APRI (AUC, 0.77) for in-hospital mortality in patients with SFTS was superior to that of NLR (AUC, 0.65), PLR (AUC, 0.54), and SII (AUC, 0.58). In conclusion, APRI is a reliable and easy-to-use serum biomarker for predicting survival outcomes in patients with SFTS.

Metabolomics has emerged as an easy-to-use assay for understanding the interaction between the host and pathogen at the small-molecule level (28). Metabolomics has been applied to various infectious diseases, including SFTS. Zhang et al. (29) detected changes in urinary metabolites in 88 patients with SFTS and found that phenylalanine-tryptophan metabolism is involved in the pathogenesis of SFTS. Li et al. (30) conducted a metabolomics analysis and found that arginine was substantially downregulated metabolite in SFTS cases compared to healthy controls, indicating that arginine metabolism, mediated by arginase, plays an essential role in SFTS. Moreover, a recent study compared the metabolic alterations between patients with SFTS treated with intravenous immunoglobulin and those who were not, identifying the top three substantial metabolites (L-kynurenine, taurohyocholic acid sodium salt, and tauro-alpha-muricholic acid sodium salt) between the two groups (31). KEGG analysis identified tryptophan metabolism as the most important pathway. In our study, we also utilized metabolomics to explain the potential mechanism underlying the poor prognostic significance of APRI in SFTS. Our metabolic analysis revealed that 2,5-diamino-6-(5-phospho-D-ribitylamino)pyrimidin-4(3H)-one, coenzyme B and rutacridone epoxide were the top three metabolites between the two groups. KEGG analysis identified alanine, aspartate, and glutamate metabolism as the most important metabolic pathways. Dysfunction in aspartate metabolism can lead to abnormal alterations in AST and APRI levels. We also found that tryptophan metabolism was the third most important signaling pathway in SFTS, which aligns with findings from previous studies.

The present study has three limitations. First, this was a retrospective cohort study of SFTS, and all patients with SFTS came from Wuhan Union Hospital. The retrospective nature of the study introduces potential confounding indices; however, we utilized PSM analysis between the low and high APRI groups to minimize confounding factors, reinforcing trust in our conclusion. Second, we only used APRI on admission to predict the survival outcome of patients with SFTS, so we could not assess the dynamic changes in APRI during the course of SFTS due to unavailability of data. Finally, although we identified substantial metabolites between the low and high APRI groups, we did not further explore the cellular or molecular mechanisms of the metabolites involved in the pathogenesis of SFTS. Further research is needed to validate the prognostic significance of APRI and investigate the detailed mechanism of APRI to elucidate the pathogenesis of SFTS.

## 5 Conclusion

Our clinical research indicates that APRI score exhibited a good predictive value for the survival outcome of SFTS patients. Metabolic analysis revealed that the significant metabolites were mainly enriched in alanine, aspartate and glutamate metabolism pathway. APRI greater than 35.3 could provide the medical workers the information for predicting the early-risk stratification of SFTS patients, and thus take intensive medical care and aggressive treatment.

## Data Availability

The original contributions presented in the study are included in the article/supplementary material. Further inquiries can be directed to the corresponding authors.
